# Health reform in Mexico: governance and potential outcomes

**DOI:** 10.1186/s12939-019-0929-y

**Published:** 2019-02-07

**Authors:** Jorge L. León-Cortés, Gustavo Leal Fernández, Héctor J. Sánchez-Pérez

**Affiliations:** 1 Departamento de Conservación de la Biodiversidad, El Colegio de la Frontera Sur, Carretera Panamericana & Av. Periférico Sur S/N, 29290 San Cristóbal de Las Casas, Chiapas Mexico; 2 Network GRAAL (Research Groups for America and Africa Latins), San Cristóbal de Las Casas, Chiapas Mexico; 3 Unidad Xochimilco, División de Ciencias Biológicas y de la Salud, Universidad Autónoma Metropolitana, Calzada del Hueso 1100, Col. Villa Quietud, Delegación Coyoacán, 04960 Mexico City, Mexico; 4 Departamento de Salud, El Colegio de la Frontera Sur, Carretera Panamericana & Av. Periférico Sur S/N, 29290 San Cristóbal de las Casas, Chiapas Mexico

**Keywords:** Public policy, Universality, Health care, Outsourcing

## Abstract

Adopting key mechanisms to restructure public policy in developing countries is a crucial political task. The strengthening of infrastructure of health services, care quality, monitoring and population health; all might contribute to assuring the functionality of a national system for health monitoring and care. Over the last decades, the Mexican government has launched wide-ranging political reforms aiming to overcome socioeconomic and environmental problems, namely health, education, finances, energy and pension. The proposed (but yet not implemented) health reform in Mexico during E. Peña Nieto’s administration (2012–2018) pretended an adjustment in Article 4 of the Mexican Constitution to compact medical care and reduce the State’s responsibility to a provision of minimum health packages for the population. Here we use a simple analytical model to describe and interprete the concepts of context, process, actors and content and the outcome of three of the most important resulting components of this intended reform i.e. universality, basic packages, and ‘outsourcing’. In light of the start of the Mexico’s new federal administration, we argue that, if not properly defined by all actors, the implementation of such structural health reform in Mexico would precipitate a model of private/public association exacerbating a crisis of political representation, human rights, justice and governance.

## Background

Mexico has been considered as one of the countries with highest levels of population inequality in the world [1], but see: [2]. Recent reports suggest that Mexican population is in a period of epidemiological transition i.e. suffering from infectious diseases to chronic degenerative diseases [3, 4]. Over the last three decades, the Mexican goverment has launched wide-ranging political reforms aiming to overcome socioeconomic and environmental problems, namely health, education, finances, energy and pension. After the announcement of the health “reform” in 2012, E. Peña Nieto’s federal administration (2012–2018) proposed an adjustment in Article 4 of the Mexican Constitution, intending to compact medical care and reducing the State’s responsibility to a provision of minimum health packages for population. The proposed health reform would envisage operating upon unique financing (mostly coming from taxes and joint resources of the main governmental health institutions), to offer basic health packages. If implemented, the primary effects of the reform would force people to overcome major health problems from private insurance and free choice of user services. However, the backbone of these intended health and social security policies lacks of appropriate operational tools [5], and hence its implementation would remain essentially technocratic. Can Mexican people reach universality with a minimum package of health services? Does the implementation of a health reform imply more benefits and better resolution of health services?

**Fig. 1:**
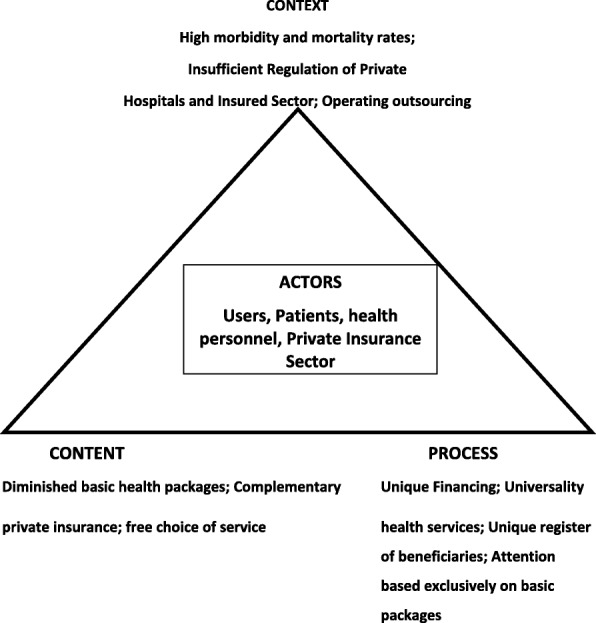
A model for Mexican policy analysis (see text)

In this paper we use a model for public policy analysis [6] as a framework to assess the potential success of a structural health reform in Mexico. Since new forms adopted by public management have been essential issues in the public policy debate [7–9], we consider this framework as an importan guideline to incorporate the concepts of context, process, actors and content, for interpreting the potential outcome of three of the most important components of the intended health reform in Mexico (as stated by Peña-Nieto’s administration, Fig. 1), i.e. universality, basic packages, and ‘outsourcing’. With the basic components of the intended reform, we then formulate an interpretation of the challenges confronted by the Mexican health system, and discuss these in light of the Mexico’s new president setting country’s course. We anticipate that, if not properly defined by all actors, the implementation of such structural health reform in Mexico would precipitate a model of private/public association health, potentially exacerbating a crisis of political representation, human rights, justice and governance.

## Universality

Universality is a sophisticated concept drawn up by decision-makers. The World Health Organization (WHO) considers the concept of “universal health coverage” [10], but in plain citizen’s language, universality not only considers complete health coverage, but people’s heartfelt simple request: an immediate improvement of health services! Based on the recent WHO definition, Mexican Health Foundation (FUNSALUD [11]) unrolled a version of universality that –seemed- to operate in the opposite direction to the public needs [12]. Immediately after Mercedes Juan was appointed as Health Secretary (2012–2016), she activated an agenda that shifted the offer made during the Mexican President Peña Nieto’s campaign and the insights of “universality” from the former director of the Mexican Institute for Social Security (IMSS), Santiago Levy. A new operating agenda would offer an extremely limited “universality” scheme, not covering for chronic degenerative diseases such as renal insufficiency, some types of cancer, nor for other important diseases with high rates of morbidity and mortality among Mexican people. This emerging agenda published between December 2012 and April 2014 considered, among other objectives, the inclusion of a unique fund raised by general taxation (raised from the main health public institutions: Ministry of Health –SSA-, Social Security Mexican Institute –IMSS-, and Social Security and Services Institute for State Workers -ISSSTE), and a strong presence of the private insurance sector – which implied a subrogation for services, and freedom of choice and prioritization of operations and illnesses [11].

The main problem of health in global population terms is not only the coverage issue, but also the quality of health services and the types of diseases and health problems covered. Such health issues include prevention, diagnosis and treatment, as well as attention for major causes of morbidity and mortality, particularly contagious diseases (i.e. respiratory infections, diarrheal, tuberculosis), malnutrition, diabetes, heart diseases, maternal mortality, chronic renal failure, and for all types of cancer [13]. By contrast, the intended Mexican health reform was proposed in exactly the opposite direction, i.e. a reduction in the number of health interventions (< 400 services), whereas IMSS’ affiliated people currently have protection at least against more than 13,000 health problems (Table 1).

**Table 1:** Key features describing the Mexican healthcare system –current and proposed modifications for the intended health reform during the Peña-Nieto’s Mexican administration (2012–2018)

	Current	Proposed
Universality	Various types of access to health services, according to the employer/employee relationship, i.e. state employees (federal level) are covered by ISSSSTE; those who work in any of the 32 Mexican states have local-state health services (e.g. for Chiapas Mexican state employees are attended by ISSTECH); Ministry of National Defense (Mexican Army, Mexican Air Force) and Ministry of Navy employees are attended by their own health services; PEMEX (Mexican Petroleum) employees are attended by their internal medical services. Senior Mexican government employees (e.g. ministers, congressmen, judges), all have major private medical insurance paid by the state. People formally employed in the private sector are attended by IMSS medical services; some private companies (such as banks) offer employees insurance for major medical expenses. Similarly, people who can afford to pay may arrange private insurance. Finally, the general population with no formal employment, e.g. employed by outsourcing, or unemployed people are not eligible for any of the medical benefits described above. However, “Popular Insurance” (“Seguro Popular”), represents an option for these people. This insurance allows people to be eligible for a “Unique Catalog for Health Services”), that includes around 384 medical surgeries. An important proportion (33.2%) of the Mexican population are not eligible to any of the above medical schemes; instead they are being offered health services through health programs of the Secretary of Health (SSA) or IMSS-‘Prospera’ or ‘Popular Insurance’ (CONEVAL, 2010). For both SSA and IMSS, medical attention is structured in three levels of care: first level (via health centres or family medicine units), second level (via general hospitals, which provide internal medicine, gynaecology obstetrics, paediatrics and general surgery) and third level services (special care by SSA is offered via national institutes of health -cardiology, nutrition, and orthopaedics, among others). In the case of IMSS hospitals, 21 Century National Medical Centre Hospital, “The Race”, Orthopaedics Hospital and Trauma Hospital. Moreover, it is becoming popular to be attended by practitioners employed at pharmacy stores (e.g. “Farmacias del Ahorro” and “Similares”), which provide basic medical consultation at relatively affordable cost. Also, in rural areas, indigenous population frequently use traditional medicine.	Introduction of Universal Health Insurance (purchased by individuals with financial support from taxation by consumers) to finance some services; general taxation to remain as the core financing mechanism. Multiple competing private insurers for financed services. Money follows the patient for financed hospital services. It includes the possibility of public-private partnerships to stimulate investments that allow expanding the provision of health services
Basic packages	Health services provided by government institutions such as IMSS, ISSSTE, SEDENA, Semar, and Pemex, cover all kind of diseases and health problems of their beneficiaries. Health services provided by government institutions such as SSA (Ministry of Health) and IMSS-Prospera, and the 32 State Systems of Health (Sistemas Estatales de Salud, SESA), provide first, second and third level services of care (most of the time directly and sometimes, through subrogated contracts), mainly via “Seguro Popular” (Popular Insurance). They include a provision of basic programs established in the so-called “Primary Health Care”, in which 12 preventive programs include immunizations, baby setters, and prenatal care.	Creation of a “single standard that impacts on the efficiency of money-spending and on saving resources”. Degrading the “right” to health. Standardizing treatment protocols and various institutions to apply rates that “explicitly” refer to a basic package. Increasing a rationalized list of interventions as “explicit” basic packages (“Universal Catalogue of Health Services” and Catastrophic Fund). Creating an “office” to oversee and check the “enforceable” condition of the “explicit” minimum packages.
Outsourcing	In recent years, this type of contract in the country has become more frequent. The labour reform promoted by the Mexican government aims to regularize this type of recruitment scheme, although under the current legislation. It fails to incorporate disease working tables or valuation of permanent disabilities. It increases the intensity of working day hours with fewer rights and salaries; minimum social protection, pensions and low health services are also increasingly compacted.	Increasing the intensity of working day hours with fewer rights and salaries, minimum social protection, pensions and low health services increasingly compacted, resulting in a precarious quality of jobs.

Perhaps the gap between reality and former President Peña Nieto’s perspective suggests a missing message on the real status of health and social security for the Mexicans. Instead of acknowledging the documented epidemiological transition of Mexican people and the need to design an ad hoc health program i.e. for a population suffering from infectious diseases to chronic degenerative diseases see [3, 4], he played around with a demagogic message based upon a supposedly “universal security system”, followed by an imposition of an outside agenda [11]. As a result, the government health reform ended up to be a non-track project, with lack of sociopolitical representation, human rights and justice, corrupt, and unpunished, all of which have triggered a severe social crisis catalized by insufficient health services with low resolutive capacity and important resource shortages.

## Basic packages

Eduardo González-Pier (Under-ministry of integration and development of the health sector) announced major adjustments to the reform at the Social Security Week organized by the Social Security Commission of the Mexican Senate. González-Pier suggested Mexican senators to simplify the reform to an amendment in Article 4 of the Mexican Constitution, and particularly to minimize the “right” to health [14]. Upon this new constitutional basis, Mercedes Juan (replaced by Jose Narro Robles as Health Minister in February 2016) proposed to enter “explicit” health services, by minimizing health operations and “precise conditions for access” to social security institutions, namely IMSS, ISSSTE, SEDENA (Secretary of National Defense), SEMAR (Mexican Navy) and PEMEX (Mexican Petroleum), i.e. the operating ‘minimum health basic packages’. In practical terms, ‘minimun health basic packages’ would seek: a) to standarize treatment protocols and force various institutions to apply rates that “explicitly” refer to a package of services; b) to gradually increase a rationed list of operations; c) to implement an “office” to oversee and check the “enforceable” condition of the “explicit” minimum packages. This ad hoc “office” would be in the capacity to fine any institution for not providing health services; and otherwise would assist patients needing (paid) relocation to a private institution. 4) To integrate a National Universal Health Commission with all institutions, including the Ministry of Finance, with the task of running minimal rations of basic packages and determining costs and agency fees (a so-called ‘portability’ for which patients could choose the type of health service and required attention [14]).

On the other hand, over the course of Peña-Nieto’s administration, there has been a special mention of strong support, particularly from the Ministry of Finance, and mainly seeking to strengthen the various strategies for Medical Tourism [15–17]. This has involved strengthening the logic of attracting investors to build clusters of competitive services, prices, professional skills and taking advantage of the strategic location of Mexico, just as occurs in other countries i.e. Thailand and Cuba (see below). In the context of public health policy, medical tourism opens the horizon for the provision of comprehensive services, with a national basis, and for those with ability to pay, which contrasts with the stepping down of the “prioritization” via the basic minimal-packages for those (the great majority) unable to pay [18].

## Outsourcing

Between the first adjustment and the latest version of Peña Nieto’s reform, a strongest regional medical mobilization was recorded (June 22, 2014 and 2016): ‘soymédico17’ – I’m a physician17 [19, 20]. Just after two months of its latest release, a first major statement of disagreement was recorded from the nursing sector, along with social workers and therapists [21]. In both cases, these vast mobilizations included a clear and abundant representation by young medical practicioners. Mobilizations represented a massive complaint against the harmful impacts of Peña Nieto’s reform for young people [22]. As a result, in 2016 numerous mobilizations by health personnel in different parts of Mexico claimed both minimal medicine supplies and labor regularization.

In sum, the intended ‘new’ health reform would affect health and social security: new rules and rates of hiring combined with impact on the taxation upon young people would compromise benefits from IMSS and ISSSTE: the so-called ‘outsourcing’. By definition, outsourcing does not incorporate labor-caused diseases or the valuation of permanent disabilities, which instead could be reduced to an administrative classification. Rather outsourcing supposes to increase the intensity of working day hours with fewer rights and lower salaries, minimum social protection, pensions and decreasing health services. The paradigm of “modernity” upon health with the intended Peña Nieto’s reform, embarked on very long detour, and precipitated extremely low wage costs and a neglected environmental for investment, i.e. the creation of jobs with monthly salaries of around 150 USD, with a minimum social protection –the basic “story” of neoliberalism!

In this regards, we think two different strategies with different impacts have become a crucial issue for public policy on health and social security: ‘sufficiency parameters’ for trained and motivated health workers [or a recapitalization of the health sector: a minimum –practicioner- capacity to provide a timely and decisive service WHO/PAHO], and the facilities (i.e. basic infrastructure) to provide proper care. These parameters would promote the definition of “universal health coverage” [10, 13, 23, 24], but also confirm the huge structural gap between FUNSALUD’s view and the real needs of the Mexican labor sector.

All in all, the exclusive “modernity” comprises the new “rights” components in a social gradient that implies, no universal pension (or an extremely low-paid pension, if it exists), false universal unemployment insurance, and a compaction of the “right” to health to mere basic health packages, which Mercedes Juan and Peña Nieto sadly touted as “explicit assurances” (Article 4 of the Mexican Constitution).

## Conclusions

During the six years of his administration, President Peña Nieto and his team operated an erratic approach for health and social security. Their intended health reform project included a non-universal pension (Program Senior Citizen directed for 65 year-old people and older [25]), an absence of childcare facilities [26], and the extension of catalogs of the ‘Prospera’ Health Program component (12).

What then should be the major focus for Mexican health policy? In addition, what kind of universality do Mexicans need? First, a true universality requires active participation of all actors (see Fig. 1). Second, the health sector badly needs to update and expand the catalog of benefits (entitlements) to citizen health care (in line with its profile and levels of morbidity and mortality). Third, in the face of the so-called “modernity” project, a potentially sensible action would require an inclusive agenda of opinion from all different actors, as well as getting agreements to build up a common agenda towards an effective and comprehensive integral universality for the majority [23]. Fourth, we think that it is necessary to go further and demand the improvement of social health determinants since the current emphasis does not achieve an adequate standard for health care for the Mexican population.

The main challenge confronted by the Mexican health system and by other countries in which Neoliberal policies operate, would be to ensure that an important proportion of the population gain access to wide health coverage, including, access, quality, and costs [27], and a key government regulation of private non-profit entities [28, 29]. In Latin America and the Caribbean region, Cuba’s health care system has been recognized worldwide for its efficiency, even when extremely limited resources and negative effects have caused serious comercial, financial and economic blocking imposed by the United States of America since 1960. In Cuba’s model, human beings at the center of the system, with health-care standards comparable to the ones in the most developed nations. In other words, public health practices are implemented under the principle that health is a basic right and a national priority (i.e., the case of Peru; [30]), and timely access to treatment for cronic health problems, including cancer in children, breast cancer, ischaemic heart disease, HIV/AIDS and diabetes (i.e., the case of Chile; [31]).

The number of poor people in Mexico has increased from 53.3 to 55.3 millons in only three years [1], and some preventable health problems as maternal mortality [32] and Tuberculosis [33], are far from being solved (Table 1). Social health institutions such as IMSS, Ministry of Health (SSA), ISSSTE and PEMEX, face a dramatic finantial crisis. So what is left for the Mexican government in years ahead, demands tackling a failed decentralization [i.e. an effective decentralization process should consider local and federal governments priorities to identify the features of common or exclusive pathological phenomena, which in any case would allow to outline a more effective strategy for attention and service]–a daunting task that will require reconstructing a battered health sector and a degraded ‘social security’ inherited by two former Mexican presidents, Vicente Fox and Felipe Calderon.

The universality of health cannot be -but it was- masked with demagogic projects such as the launching of ‘high-quality’ hospitals in several places (i.e. Cananea, Sonora, and Chiapas, or the National Accord Obstetric Emergencies), which were formally inaugurated but soon forgotten. If implemented as proposed by the Peña Nieto administration, a health reform will exhacerbate the already limited-cover to attend illnesses not explicitly included in the so-called basic packages (i.e. more than 10,000 illnesses and health problems are currently attended by social security institutions, Table 1). Similarly, outsourcing contracts for young people would imply an absence of social lending and low salaries, which in turn would amplify the need for health services; and to that end, wealthier people could only be the ones capable of hiring private services to complement health services, and hence priviledging private insurance brokers.

Overall, the limited vision of the former Mexican administration for social and health security was far from being able to attend the real needs of the population. As in other areas of social policy and governance, the Peña Nieto’s exclusive “modernization” project sank precipitously with high costs for the majority of Mexicans. It remains to be seen whether the Mexico’s new administration would be capable of implementing an effective strategy for the health sector, a strategy where changes mean improving actions, programmes and sufficiency parameters of trained and motivated health workers, as the means to provide a truly “universal health coverage”.
